# Jellyfish Collagen: A Biocompatible Collagen Source for 3D Scaffold Fabrication and Enhanced Chondrogenicity

**DOI:** 10.3390/md19080405

**Published:** 2021-07-22

**Authors:** Zara Ahmed, Lydia C. Powell, Navid Matin, Andrew Mearns-Spragg, Catherine A. Thornton, Ilyas M. Khan, Lewis W. Francis

**Affiliations:** 1 Medical School, Swansea University, Singleton Park, Swansea SA2 8PP, UK; zaraahmed109@gmail.com (Z.A.); L.C.Powell@swansea.ac.uk (L.C.P.); navid_matin2004@yahoo.com (N.M.); C.A.Thornton@swansea.ac.uk (C.A.T.); I.M.Khan@swansea.ac.uk (I.M.K.); 2 Jellagen Ltd., Unit G6, Capital Business Park, Parkway, Wentloog Industrial Estate, Cardiff CF3 2PY, UK; andrew@jellagen.co.uk

**Keywords:** osteoarthritis, articular cartilage, jellyfish collagen, MACI

## Abstract

Osteoarthritis (OA) is a multifactorial disease leading to degeneration of articular cartilage, causing morbidity in approximately 8.5 million of the UK population. As the dense extracellular matrix of articular cartilage is primarily composed of collagen, cartilage repair strategies have exploited the biocompatibility and mechanical strength of bovine and porcine collagen to produce robust scaffolds for procedures such as matrix-induced chondrocyte implantation (MACI). However, mammalian sourced collagens pose safety risks such as bovine spongiform encephalopathy, transmissible spongiform encephalopathy and possible transmission of viral vectors. This study characterised a non-mammalian jellyfish (*Rhizostoma pulmo*) collagen as an alternative, safer source in scaffold production for clinical use. Jellyfish collagen demonstrated comparable scaffold structural properties and stability when compared to mammalian collagen. Jellyfish collagen also displayed comparable immunogenic responses (platelet and leukocyte activation/cell death) and cytokine release profile in comparison to mammalian collagen in vitro. Further histological analysis of jellyfish collagen revealed bovine chondroprogenitor cell invasion and proliferation in the scaffold structures, where the scaffold supported enhanced chondrogenesis in the presence of TGFβ1. This study highlights the potential of jellyfish collagen as a safe and biocompatible biomaterial for both OA repair and further regenerative medicine applications.

## 1. Introduction

Osteoarthritis (OA) is a common degenerative disease that affects the entire synovial joint with articular cartilage sustaining the most critical damage [1]. OA significantly impairs the quality of life of 8.5 million people in the UK due to chronic pain and limited joint movement [2]. The prevalence of OA is expected to rise with increasing life expectancy [3], resulting in a high economic burden for health care providers. The National Health Service (NHS) and wider healthcare systems in the UK spent £10.2 billion treating both OA and rheumatoid arthritis in 2017, with a cost of £118.6 billion projected over the next decade. In addition, these diseases cost the UK economy £2.58 billion a year through 25 million lost working days in 2017 [4]. More effective treatment strategies are therefore urgently needed to combat patient morbidity resulting from OA.

Adult articular cartilage is formed of an integral extracellular framework primarily composed of collagen, which accounts for approximately two thirds of its dry mass [5,6]. Collagen belongs to a family of fibrous and non-fibrous proteins and contributes to cartilage tissue architecture and strength, while also promoting cell growth, differentiation and migration [7,8]. OA is characterised by degradation of the cartilage extracellular matrix (ECM) caused by aging, abnormal mechanical loading, nutritional deficiencies or blunt trauma and injury [9]. Therefore, collagen-based surgical repair strategies, such as matrix assisted autologous chondrocyte implantation (MACI), have been increasingly implemented to effectively repair damaged cartilage tissue [10,11,12]. Collagen is an ideal biomaterial to produce scaffold matrices for MACI due to its material properties of high mechanical strength, flexibility, biocompatibility and controllable biodegradability [13]. Currently, a range of collagen-based scaffold products are commercially available for MACI procedures, such as MACI, Novacart3D and CaReS [14] however, these collagen sources have been derived from mammals with particular focus on bovine and porcine origins [15]. Recently, safety issues have arisen with the use of collagen from mammalian sources due to the outbreak of zoonotic diseases such as bovine spongiform encephalopathy (BSE), other transmittable spongiform encephalopathies (TSEs) and potential viral vectors which have potential to be transmitted to humans. Also, ethical and religious restrictions and the discovery that approximately 3% of the population is allergic to bovine material [16], further complicate the use of mammalian derived collagen in surgical interventions.

More recently, collagen derived from alternative marine sources, such as fish, jellyfish and sea urchins, have been investigated to overcome ethical and immunological issues surrounding mammalian collagen [17]. Particular interest has been paid to jellyfish as their anatomy is largely composed of collagen and the increase in water temperatures due to global climate change have caused seasonal blooms resulting in a plentiful supply [18]. Recent studies have extracted collagen from jellyfish [19,20,21], and shown it to resemble mammalian collagen types I, II and V. Jellyfish collagen has also been referred to as collagen type 0 [20] due to its similarity to multiple collagen types and because of the jellyfish ancient biological and chemical lineage [22,23,24,25]. Jellyfish collagen has demonstrated the capacity to maintain cellular phenotype without dedifferentiation and support differentiation of embedded cells [26]. Importantly, researchers have demonstrated that jellyfish collagen is non-toxic, maintains a higher cell viability of osteoblasts and fibroblasts compared to bovine collagen [21,27], induces a long-term anti-inflammatory macrophage response in vivo [20] and can be used as a nanoresevoir for growth factors to aid in the regeneration of cartilage [28]. These studies demonstrate that jellyfish are a potential source of collagen for MACI in OA repair and further regenerative medicine applications.

This study was performed to explore the suitability of *Rhizostoma pulmo* jellyfish collagen for biocompatible scaffold fabrication and provide proof of concept for 3D chondrogenicity. The material properties of jellyfish collagen scaffolds were characterised in terms of pore size, thermal stability and biodegradability. The jellyfish collagen scaffolds were tested in vitro for their immunogenic potential using healthy human blood and the response compared against bovine and rat tail collagen, as well as bovine gelatin. Assessment of jellyfish collagen scaffolds in supporting chondrogenesis was also determined by culturing bovine chondroprogenitor cells in chondrogenic media for 21 days before biochemical and histological analysis for cell DNA, glycosaminoglycan (GAG) content and collagen orientation of the scaffolds was performed.

## 2. Results

### 2.1. Structural Characterisation of Jellyfish Collagen Scaffolds

**Figure 1:**
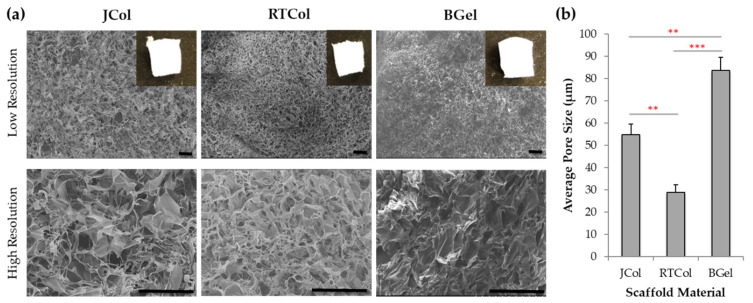
Characterisation of collagen scaffolds using scanning electron microscopy. (**a**) Low magnification and high magnification SEM images of the JCol, RTCol and BGel scaffold architecture with 1% EDC cross-linking treatment. Scale bars equal to 200 μm. (**b**) Average Pore Size (μm) calculated from the high magnification SEM images shown with standard deviation error bars (minimum of three independent repeats). Statistical significance shown according to Mann Whitney U test (** *p* < 0.01; *** *p* < 0.001).

To assess *R. pulmo* collagen for use in matrix-assisted repair strategies, extracted collagen was manufactured into sponge scaffold constructs (JCol) and compared with research grade rat-tail collagen (RTCol), bovine collagen (BCol) and clinical grade bovine gelatin (BGel) a source of denatured collagen. The freeze-dried collagen scaffolds (4 mg/mL) were crosslinked with 1-ethyl-(3-3-dimethylaminopropyl) carbodiimide hydrochloride (EDC; 1% *w*/*v* in 80% ethanol) for 90 min to form cylindrical constructs, measuring approximately 3 mm in width and 5 mm in height (Figure 1a inset; top surface of the scaffolds). The microstructure of the JCol scaffolds, as assessed by scanning electron imaging (SEM), was comparable with the RTCol and BGel scaffolds, showing interconnected pores with boundaries that are defined by sheet-like structures of dense afibrillar collagen (Figure 1a).

Scaffold pore size and pore interconnectivity can affect cell attachment, migration and media exchange [29], all factors which are crucially important in matrix-assisted cartilage repair. Scaffold pore sizes measured from the SEM images revealed that JCol scaffolds have an average pore size of 54.8 ± 4.8 µm; significantly greater than RTCol sponges (28.8 ± 3.4 µm; *p* < 0.01) and, as expected, significantly smaller than the control BGel sponge structures with an average pore size of 83.7 ± 5.9 µm (*p* < 0.01, Figure 1b). Therefore, the average pore size for JCol scaffolds falls between the pore sizes of the mammalian collagen sourced scaffolds, with comparable pore shape and homogeneity throughout the whole scaffold.

Sequence alignment of jellyfish collagen peptides to human COL1A1 and COL3A1 collagen genes [30] was performed using Clustal Omega (data not shown) and AbDesigner to determine the nucleotide positions with high similarity to *R. pulmo* for functional relevance [31]. Supplementary Figure S1 highlights the regions of sequence similarity between human COL1A1 and COL3A1 and *R. pulmo* peptides. The sequences are shown to localise to the mature collagen α1 chain which forms part of the triple helical region (nucleotide positions 998 to 1015 and 1070 to 1080 for COL1A1 and 926 to 960 for COL3A1). This data reveals that *R. pulmo* collagen displays similarity to human mature collagens type I and type III [30].

### 2.2. Stability and Purity of Jellyfish Collagen Scaffolds

**Figure 2:**
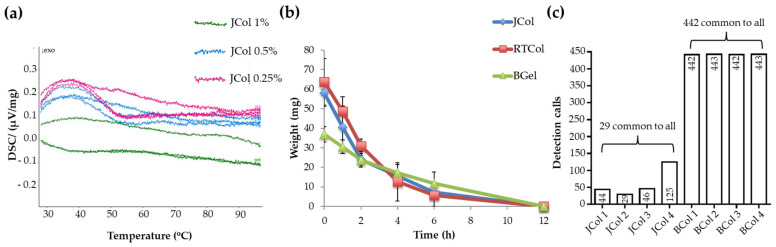
Stability and purity of collagen scaffolds. (**a**) Differential scanning calorimetry (DSC) of JCol scaffolds crosslinked with 0.25%, 0.5% and 1% *w*/*v* EDC. (**b**) Collagenase treatment of JCol, RTCol and BGel scaffolds (1% *w*/*v* EDC), where the scaffolds were weighed regularly to assess resistance to degradation. (**c**) miRNA detection in four samples of JCol and BCol, where the number of detected human miRNA sequences for each sample is listed within the respective bar column, while the numbers of consistently detected human miRNA sequences are shown in brackets for each sample type.

Scaffolds produced for regenerative medicine should possess stability against thermal degradation [32]. Differential scanning calorimetry (DSC) can determine the resistance of biological macromolecules to thermal degradation by measuring the differential heat flow from the calorimeter, as the samples are heated linearly over a period of time. DSC has previously been used to identify biopolymer melting, lipid-protein interactions and conformational changes of proteins [33] and has also been used to assess the efficiency and extent of crosslinking in scaffolds [34]. The thermal stability of JCol, RTCol and BGel scaffolds, crosslinked with 0.25, 0.5 and 1% EDC, were assessed from room temperature to 100 °C (Figure 2a and Figure S2).

Thermal analysis revealed that JCol and RTCol constructs crosslinked with 0.25% and 0.5% EDC exhibited peaks located at approximately 37 °C, whilst the scaffolds crosslinked with 1% EDC demonstrated a much reduced but broader peak located between 50–60 °C (Figure 2a and Figure S2). This data indicated that JCol and RTCol scaffolds cross-linked with 1% EDC demonstrated a higher thermal stability over the temperature range of 30–90 °C. Conversely, the BGel constructs crossed-linked with 1% EDC demonstrated the greatest change in thermal energy, displaying a peak at 37 °C, while the constructs crossed-linked with 0.25 and 0.5% EDC revealed a much reduced but broader peak at approximately 37 °C (Figure S2). Therefore, JCol constructs crossed-linked with 1% EDC were chosen for the rest of the study.

Scaffolds designed for regenerative medicine applications must also fulfil the requirement of being biodegradable to enable adequate remodelling of the damaged tissue by the host [35]. In vivo, collagenases such as matrix metalloproteinases (MMPs) are responsible for collagen degradation by initially unwinding the triple helix of collagen, making the molecule labile to digestion by a host of other enzymes. As bacterial collagenases operate under similar principles, they can be used to investigate degradation in vitro [36]. Scaffolds fabricated from RTCol, JCol and BGel, crosslinked with 1% EDC, were incubated with bacterial collagenase and scaffold weight was monitored to assess degradation over time. JCol and RTCol scaffolds demonstrated similar reductions in weight with time, until no material remained after 12 h (Figure 2b), indicating similar properties of biodegradability between the collagen samples. BGel constructs displayed a much slower rate in weight reduction however, no BGel remained after 12 h which was comparable to RTCol and JCol samples.

Mammalian collagen sources are often linked with a pathological risk for transmitted disease, and the purification processes required for clinical use of the biomaterial are currently challenging [16,20]. Jellyfish collagen in contrast has a simple physiology, with a collagen content of more than 60% [20,24]. A standardised assay for collagen purity is testing extracts for miRNA, where xenogenic miRNA may cause adverse reactions in patients and affect biocompatibility. Detection of miRNA in JCol samples revealed only 29 human miRNA sequences common to all four samples, which was much lower than the 442 common human miRNA sequences detected in BCol (Figure 2c). This assay indicates that JCol possess a higher purity when compared to mammalian collagens.

### 2.3. Platelet Activation in Response to Jellyfish Collagen Scaffolds

Collagen scaffolds are required to be screened for their immunogenicity before clinical use [37]. The accepted methodology for this process is centred on complex and expensive in vivo models such as murine or rat models [38]. Recently, Flaig et al. [20] demonstrated in murine models that JCol is biocompatible and appropriate for bone regeneration. In addition to this proof of concept study, an initial understanding of the human immune response can be provided using healthy blood donors with active immune cell components, so called in vitro immunogenicity [39]. Radley et al. [40] showed that in vitro immunogenic assessment of biomaterials could be achieved through flow cytometry of a panel of leukocyte and platelet markers following the incubation of human blood with the desired material. The immunogenic response elicited by JCol, RTCol, BCol and BGel scaffolds was determined by assessment of the collagen’s effect on platelet activation, leukocyte activation and cell death. A series of CD markers were identified and used to gate platelet, neutrophil, monocytes and T cells by cell number (CD42b, CD11b, fMLPr and DRAQ7) and/or median fluorescent intensity (CD62L).

**Figure 3:**
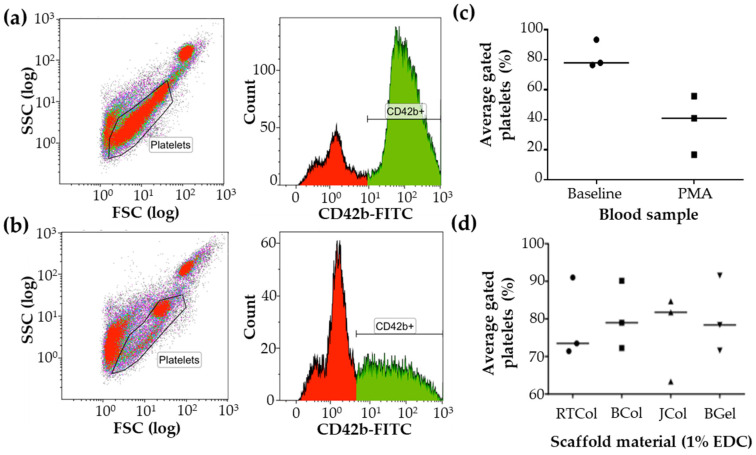
Platelet activation in the presence and absence of PMA and collagen scaffolds. (**a**) Blood from three healthy donors were analysed by flow cytometry for CD42b expression, shown as a representative scatter plot and histogram from a single healthy volunteer, and (**b**) compared to PMA stimulated blood (2 h incubation). (**c**) Analysis of flow cytometry data for unstimulated and PMA stimulated blood samples were obtained with proprietary flow cytometry analysis software (Kaluza, version 1.2, Beckman Coulter, High Wycombe, UK). (**d**) Whole blood was incubated for 2 h with JCol, BCol, RTCol and BGel samples (1% *w*/*v* EDC cross-linker treatment) and platelet activation was measured. All data shown are individual values plotted as a scatter plot with median values depicted as a line.

Blood from three healthy donors were analysed using flow cytometry for CD42b expression to indicate platelet activation. PMA treatment was used as a positive control to induce platelet activation and aggregation (resulting in shielding of CD42b in human blood models) [41], which is depicted as platelet movement out of the predefined gates (Figure 3a,b). Even though there was considerable donor variation in the data (Figure 3c,d), the median value of % gated platelets obtained with untreated blood was approximately double the value obtained with PMA treatment (Figure 3c). Following whole blood incubation with the collagen scaffolds, JCol exhibited a median value of % gated platelets that was comparable to untreated blood and the other scaffold materials (BCol, RTCol, BGel). This data indicates that JCol scaffolds do not induce platelet activation.

### 2.4. In Vitro Innate Immune Response to Jellyfish Collagen Scaffolds

**Figure 4:**
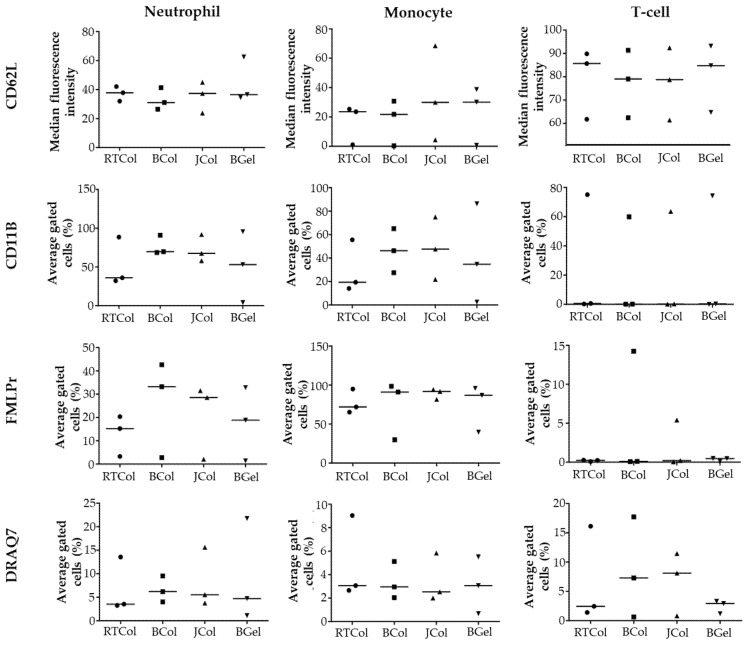
Leukocyte activation and death in the presence of collagen scaffolds. Whole blood (obtained from three donors) was incubated for 2 h with JCol, BCol, RTCol and BGel scaffolds (1% *w*/*v* EDC cross-linker treatment). Specific neutrophil (CD15+), monocyte (CD14+) and T cell (CD3+) activation was detected through staining with antibodies to CD62L; CD11b and fMLPr, while leukocyte cell death was detected through staining with DRAQ7. All data shown as summarised donor cohort responses following analysis by flow cytometry software, Kaluza, UK). Graphs shown are individual values shown as a scatter plot with median values depicted as a line.

The collagen scaffold samples were then incubated with healthy human blood to identify leukocyte activation markers expressed by neutrophils, monocytes and T cells. Assessment of the neutrophil activation markers revealed that CD62L expression in the blood after incubation with JCol was comparable to those of RTCol, BCol and BGel (Figure 4). Expression of the neutrophil activation markers CD11b and fMLPr after incubation with JCol were also comparable to BCol, however, the values of RTCol and BGel appeared to be lower. There was considerable donor variation within the data, so the significance of this lower neutrophil activation marker expression could not be assessed. These results indicate that JCol is comparable to BCol in neutrophil activation within human blood. JCol was also comparable with the other collagen scaffolds (RTCol, BCol and BGel) in DRAQ7 expression which indicated low levels of neutrophil death.

Assessment of monocyte activation markers revealed that CD62L and fMLPr expression after incubation with JCol was comparable to BGel, RTCol and BCol (Figure 4). CD11b expression for JCol scaffolds also appeared to be comparable to BGel and BCol, with only RTCol appearing to exhibit lower levels. The data indicates that JCol is comparable in monocyte activation when compared to the other scaffold materials. The levels of monocyte death after incubation with JCol were also comparable to the other scaffold materials.

Assessment of T-cell activation markers revealed that CD62L and CD11b expression after incubation with JCol was comparable across all the scaffold materials. Due to the multi-parameter staining strategy employed during flow cytometry, fMLPr expression data is available however, fMLPr expression is negligible as expected. This data indicates that JCol does not increase T-cell activation when compared to the other scaffold materials. The levels of T-cell death after incubation with JCol and BCol appeared to be higher than that of RTCol and BGel, however, JCol and BCol were comparable to each other in terms of T-cell death.

### 2.5. Cytokine Release Profile in the Presence of the Jellyfish Collagen Scaffolds

Cytokines are key modulators in inflammation and can be categorised as either pro-inflammatory or anti-inflammatory [42]. They are released upon activation of immune cells, where release is regulated in cascades responding to an initial inflammatory response followed by another cascade in order to cause the reaction to subside [43].

**Figure 5:**
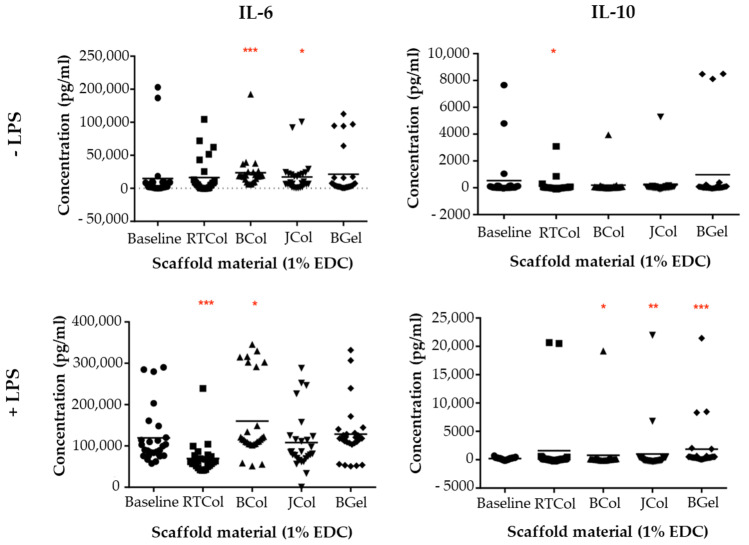
Cytokine release profile in the presence of scaffolds. Whole blood samples incubated with JCol, BCol, RTCol and BGel scaffolds in the presence and absence of LPS were subjected to ELISA to obtain concentrations of IL-6 and IL-10 released. All data shown is the standard deviation from a minimum of 3 independent biological repeats, statistical significance shown according to Mann Whitney U test (* *p* < 0.05; ** *p* < 0.01; *** *p* < 0.001).

Following incubation of healthy human blood with scaffolds, the release of the pro-inflammatory cytokine, IL-6, and the anti-inflammatory cytokine, IL-10 in the presence and absence of lipopolysaccharides (LPS) was assessed (Figure 5). JCol induced a significantly increased level of IL-6 (*p* < 0.05) when compared to the baseline blood samples in the absence of LPS however, BCol exhibited an even greater increase in IL-6 (*p* < 0.001). RTCol and BGel showed no significant difference in IL-6 concentration when compared to baseline samples. In the presence of LPS, there was no significant difference in IL-6 concentration after incubation with JCol and BGel when compared to baseline samples, however there was an increase in IL-6 with BCol (*p* < 0.05) but a decrease in IL-6 concentration with RTCol incubation (*p* < 0.001). 

The anti-inflammatory cytokine, IL-10, was also assessed following incubation with scaffolds (Figure 5). There was no significant difference in IL-10 concentration in the absence of LPS with JCol, BCol and BGel incubation compared to baseline samples however, RTCol exhibited significantly lower IL-10 concentrations (*p* < 0.05). When stimulated with LPS, RTCol exhibited no significant difference in IL-10 concentration when compared to baseline blood samples, whereas BCol (*p* < 0.05), JCol (*p* < 0.005) and BGel (*p* < 0.001) exhibit a significant increase in IL-10 concentration. These results indicate that JCol is comparable or even demonstrates reduced cytokine release when compared to the other scaffold materials.

### 2.6. Jellyfish Sponge Scaffolds Ability to Induce a Chondrogenic Effect

Chondrogenesis is a complex process that leads to new cartilage tissue formation. This process involves progenitor cell condensation, chondrocyte differentiation and ECM deposition [44]. Therefore, the regeneration of articular cartilage in OA patients requires a scaffold with a 3D architecture that supports cellular invasion and chondrogenic differentiation [45,46]. Alterative cell sources for cartilage repair have included stem cells due to their plasticity, availability and lack of donor morbidity [47]. However, current cell-based clinical practices for treating OA only use autologous chondrocytes [47]. In this study, we isolated cartilage-specific bovine chondroprogenitor cells [48] and seeded 5 × 10^5^ cells onto scaffolds in chondrogenic media for 21 days to determine their invasion, proliferation and chondrogenic capacity. This study was performed in the presence and absence of transforming growth factor-β1 (TGFβ1). In healthy human articular cartilage, even though TGFβ1 is present in high quantities it is not readily accessible by the chondrocytes, being sequestered by specific binding proteins. TGFβ1 is well documented as a growth factor to induce chondrogenesis, where studies show high levels of active TGF-β in OA patient joints [49,50].

**Figure 6:**
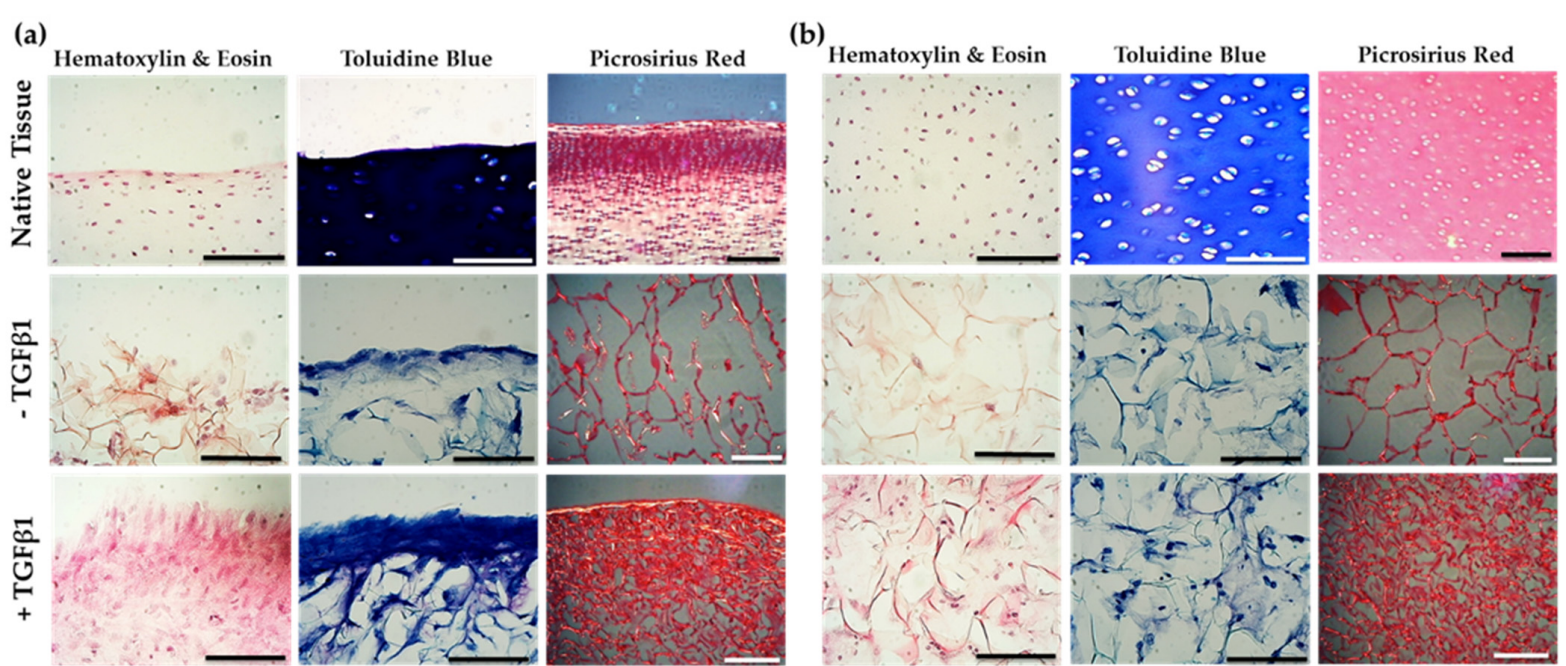
Histological analysis of cell-seeded scaffolds incubated in chondrogenic medium. Following 21 days of chondrogenic culture in the presence and absence of TGFβ1, JCol scaffolds were stained with haematoxylin and eosin (×40 magnification), toluidine blue (×40 magnification) and picrosirius red (×20 magnification) to assess for cell invasion, proteoglycan content and collagen orientation, respectively. Native immature bovine cartilage was also stained as a benchmark. (**a**) The surface and (**b**) the centre of the construct of the scaffolds were imaged using light and polarised light microscopy. Scale bars equal to 0.1 mm.

To assess cell distribution through the scaffold structure, histological examination with haematoxylin and eosin (H & E) staining was used, where cells could be unambiguously identified through the presence of nuclei (dark pink colour) [51]. Light microscopy images from the native cartilage surface (superficial zone) revealed cells possessing a flattened and discoid morphology with a closer proximity to each other when compared to cells in the deeper zones (Figure 6). In comparison, JCol scaffolds (without TGFβ1 treatment) showed a mix of both discoid and circular cells in close proximity to each other in the superficial zone of the construct, with clear staining of the scaffold’s collagen ribbons in red. In the deeper zones of the scaffold no cells were visible. In contrast, TGFβ1 (10 ng/mL) treated scaffolds displayed intense pink staining of the tissue with some circular cells seen at the surface of the scaffold, whilst in the deeper zones the cells were visible and appeared circular in shape and in close proximity to each other.

Cartilage possesses characteristically high levels of proteoglycan to aid in the initial assembly of the ECM and allow for osmotic swelling following decompression [52]. Proteoglycan degradation within cartilage is characteristic of OA, causing breakdown of both the cell-matrix communication and the fibrillar network, with biochemical changes in the tissue [53]. Toluidine blue histological staining can be used to assess proteoglycan production and deposition by the differentiated articular chondroprogenitors in scaffolds and native tissue after 21 days (Figure 6). The native tissue exhibited a dark blue stain at the surface, indicating high levels of proteoglycans, whilst in the deeper zones white areas representing chondrones indicate hypertrophic fully differentiated chondrocytes (Figure 6). TGFβ1 treated and non-treated JCol scaffolds also exhibited a dark blue band of staining at the surface which was more prevalent with TGFβ1 treatment, however, staining on the scaffolds was to a lesser extent when compared to the native tissue. In the deeper zones of JCol scaffolds, whilst the stain picks out collagen, some metachromatic staining for weaker proteoglycan deposition and cell presence, overall, the intensity of staining was decreased for the untreated scaffolds compared to TGFβ1 treated scaffolds.

The cellular deposition of collagen in tissues and scaffolds can be assessed using picrosirius red as a histological stain to identify fibrillar collagen [54]. Picrosirius red staining of native bovine tissue showed intense red staining at the surface of the tissue, indicating dense collagen distribution, with unstained areas representing cells (Figure 6). TGFβ1 treated and un-treated JCol scaffolds displayed a skeletal network of collagen ribbons both at the surface and in the deeper zones however, in the TGFβ1 treated JCol structure a denser collagen network was present indicating an additional contribution from differentiating cells.

Qualitative histological analysis of the JCol scaffolds revealed that there were increased cell numbers, increased proteoglycan levels and a denser collagen network with TGFβ1 treatment after 21 days of incubation with cartilage-specific bovine chondroprogenitor cells.

### 2.7. Biochemical Analysis of Chondrogenesis with Jellyfish Collagen Scaffolds

**Figure 7:**
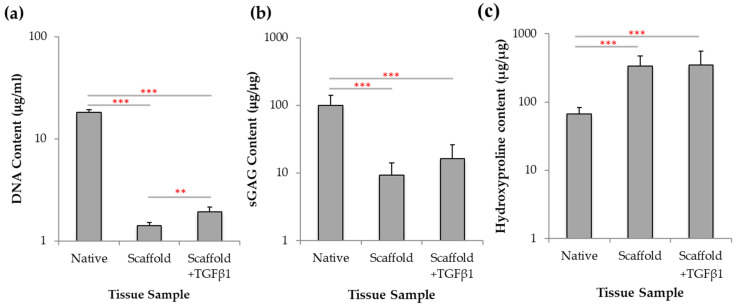
Biochemical analysis of scaffolds following in vitro chrondrogenesis. Following 21 days of chondrogenic culture in the presence and absence of TGFβ1, the JCol scaffolds were papain digested and (**a**) assessed for DNA content, (**b**) sGAG content and (**c**) hydroxyproline content, in comparison to native immature bovine cartilage. Statistical significance shown according to Mann Whitney U test (** *p* < 0.01; *** *p* < 0.001).

Biochemical analysis was undertaken of JCol scaffold cultures to investigate DNA, sGAG and hydroxyproline content with and without TGFβ1. To assess if the JCol scaffolds supported chondroprogenitor cells proliferation, quantification of DNA was obtained following 21 days incubation, using a picogreen assay. For comparison, biochemical assays were also conducted using intact, freshly isolated immature cartilage from bovine donors. Cultures treated with TGFβ1 were shown to exhibit a higher DNA content, 1.93 ± 0.22 µg/mL, when compared to those without growth factor treatment, 1.41 ± 0.10 µg/mL (*p* < 0.01; Figure 7a) however, these values are significantly less than those observed in the native tissue (*p* < 0.001).

GAGs are an important cartilage component which provide resistance to compressive loading and are involved in many other biological interactions [55]. GAG deposition was quantitatively assessed using 1,9-dimethylmethylene blue (DMMB) dye [56]. JCol cultures treated with TGFβ1 (Figure 7b) displayed an average of 16.39 ± 9.52 µg/µg of sGAG compared to 9.28 ± 4.72 µg/µg in untreated cultures, however again, these values were significantly less than those of native tissue (*p* < 0.001).

Collagen has a triple-helical structure which is characterized by the incorporation of the modified amino acid 4-hydroxyproline which aids in structural stabilisation and provides the distinctive twist to the collagen helix [57]. The amount of hydroxyproline found in cartilage collagen was quantified spectrophotometrically after 21 days of culture. No significant difference in hydroxyproline content was found between treated and untreated cultures at 350.6 ± 202.9 µg/µg and 335.3 ± 138.8 µg/µg respectively, however, these levels of hydroxyproline were significantly higher than those observed in native tissue (*p* < 0.001).

This biochemical assessment of the chondroprogenitor cells after incubation with JCol scaffolds revealed that only cell proliferation was significantly increased with TGFβ1 treatment, whilst there was no significant effect in GAG and collagen deposition without the presence of TGFβ1.

## 3. Discussion

Articular cartilage is a tissue with limited regenerative capacity, where tissue trauma can lead to the development and progression of OA [58]. Biomaterial matrix-assisted approaches, such as collagen-based scaffolds, have been clinically used to prevent further ECM degradation in OA patients by enhancing chondrogenesis and cartilage tissue production [59,60,61]. This study characterised the jellyfish *R. pulmo* source of collagen as an alternative biomaterial for 3D scaffold fabrication, which potentially could be used in MACI procedures for OA tissue repair.

Native collagen possesses an array of inter- and intra-molecular crosslinks [62] which provides tensile strength within the tissues [63]. Marine animal collagen possesses fewer crosslinks compared to mammalian collagen, due to the material’s lower content of proline and hydroxyproline, leading to less robust scaffolds and a lower denaturation temperature (29 °C) [17]. To improve the stability of the marine collagen scaffolds for clinical use, the material properties can be manipulated using a chemical crosslinker, EDC [21,32]. EDC has previously been used to form mechanically stable 3D scaffolds using jellyfish collagen [64]. In addition, EDC has been shown to be non-toxic to cells at concentrations of 1% and below [65], therefore scaffolds used in this study were cross-linked at concentrations equal to or less than 1% EDC.

Scaffolds act as a transient structure in cartilage repair, where they must possess both structural and mechanical integrity to support tissue regeneration [66]. Scaffold biomaterials must also be biodegradable to allow adequate remodelling [32] as the embedded cells produce their own native, complex and highly organised extracellular matrix. We demonstrated that jellyfish collagen scaffold pore sizes (20–150 µm) fell within the critical range for applications in cartilage tissue engineering previously reported to facilitate chondrocyte differentiation [67]. Also, jellyfish scaffolds were comparable to mammalian collagen scaffolds (bovine and rat-tail) in terms of structural morphology and pore interconnectivity. Furthermore, the jellyfish collagen displayed resistance to thermal degradation at 37 °C but allowed for collagenase degradation demonstrating essential attributes required for cartilage tissue engineering and the suitability of jellyfish collagen for clinical biomatrix-assisted OA treatments. The similarity between jellyfish collagen and human collagen in the mature collagen α1 chain, which forms part of the triple helical region, combined with the enhanced purity of the jellyfish collagen compared to bovine sources, indicates that jellyfish collagen is an attractive, alternative biomaterial for scaffold fabrication.

In clinical use, scaffolds must avoid elicitation of an immune response [66], which could lead to inflammation, fibrotic encapsulation, infection and potentially tissue necrosis [68]. Bovine collagen presents a range of contamination challenges, such as possible transmission of zoonotic diseases and also immunological considerations as 3% of the population exhibits allergic reactions to bovine-derived collagen [69,70]. Therefore, alternative sources of collagen are needed to overcome the current challenges posed by mammalian-derived collagen. Previous studies have shown that jellyfish-derived collagen immune responses were comparable to bovine collagen/gelatin and also that jellyfish collagen induced a long-term anti-inflammatory macrophage response in vivo [20,27]. Current research indicates that the structural composition of collagen plays a role in the immunogenic properties of the biomaterial [27,71]. Here, we examined the immunogenic response elicited by jellyfish collagen scaffolds by examining platelet activation, leukocyte activation and cell death. This study indicated that *R. pulmo* jellyfish collagen scaffolds do not induce platelet activation and are comparable in monocyte and T-cell activation compared to rat tail and bovine collagen/gel scaffolds. In addition, jellyfish collagen scaffolds were comparable to bovine collagen in T-cell death and were comparable to all the other scaffold constructs for neutrophil and monocyte cell death. Furthermore, assessment of the release of the pro-inflammatory cytokine, IL-6, and the anti-inflammatory cytokine, IL-10 in the presence and absence of LPS revealed that jellyfish collagen is comparable or even demonstrates reduce cytokine release compared to the other scaffold materials. These immunological assays highlight the suitability for jellyfish collagen to be used clinically, especially when bovine collagen is already used in clinic [14,15].

Articular cartilage mostly comprises of a dense extracellular matrix with a sparse distribution of chondrocyte cells. The MACI technique is a clinical operation which produces a biomimetic environment by using scaffolds for cartilage tissue repair in OA patients [72]. The procedure harvests chondrocyte cells from a non-weight bearing portion of the tissue to add to the scaffold matrix to encourage tissue formation [73], during implantation into the defect [74]. Clinical studies have shown that MACI surgery provides better cartilage repair in patients compared to those who were treated using microfracture or autologous chondrocyte implantation (ACI) [72]. Previous studies have investigated the chondrogenic potential of jellyfish collagen. *R. esculentum* jellyfish sponge scaffolds were found to redifferentiate human nasal septal chondrocytes following loss of phenotype from monolayer culture [75] and human mesenchymal stem cells (MSCs) could differentiate into the chondrogenic lineage in *R. pulmo* collagen scaffolds [28]. Also, *R. esculentum* jellyfish collagen scaffolds have also been shown to upregulate chondrogenic markers of MSCs [26] and support a higher cell viability compared to bovine collagen [20,27,28]. In our study, cell invasion by bovine chondroprogenitors was observed throughout the jellyfish collagen scaffold depth, meeting the requirement of high cell density favoured by in vitro chondrogenesis [76]. Bovine chondroprogenitors also showed enhanced differentiation within the scaffold in the presence of TGFβ1, which has previously been shown to induce mesenchymal cell condensation and initiate chondrogenesis in MSCs [50]. Histological and biochemical analyses demonstrated that jellyfish collagen scaffolds support cell proliferation and/or greater cell viability, with proteoglycan and collagen deposition associated with normal chondrogenesis. Matrix deposition in jellyfish scaffolds appeared enhanced with TGFβ1 treatment in the histological analysis, and cellular DNA content was significantly increased with TGFβ1 treatment.

In clinical use, tissue engineering scaffolds must be designed to withstand the mechanical forces that will act on the joint during patient movement which includes hydrostatic pressure, static and dynamic compression, shear and rotation [77]. However, it has been suggested that scaffold materials with a relatively low mechanical stiffness (biomimetic) could be conducive for new tissue formation [78,79]. Furthermore, studies have demonstrated that mechanical stimuli will encourage chondrogenic cells to differentiate and produce matrix, stimulating cartilage formation [80,81]. These studies demonstrate the importance of not only the mechanical strength testing of the scaffold itself but also examination of cellular interaction with scaffold mechanics and the influence of external mechanical stimuli on cellular processes within the scaffold structure. These experiments form the next stage of development of the jellyfish collagen for OA repair.

This study has proven that *R. pulmo* jellyfish collagen displays potential as an alternative, structurally compatible, cleaner, next generation collagen source, more robust for the purposes of tissue engineering in OA repair. Jellyfish collagen possesses the structural and biodegradable material properties, combined with the biocompatibility and immunogenic profiles required for clinical use for cartilage repair in OA patients.

## 4. Materials and Methods

### 4.1. Jellyfish Collagen

Purified jellyfish collagen (JCol) solution (4 mg/mL in 0.1 M acetic acid) was derived from *Rhizostoma pulmo* jellyfish and provided by Jellagen Ltd. (Cardiff, UK). The collagen was extracted using a standardised acid solubilisation procedure, with sodium acetate to remove non-collagenous materials. The purity of the JCol was analyzed using sodium dodecyl sulphate polyacrylamide gel electrophoresis (SDS-PAGE) and fourier transform infrared spectroscopy (ATR-FTIR, Perkin Elmer, Bucks, UK) [24]. In this study, the jellyfish collagen was compared to collagen used in clinical and academic applications such as rat-tail Type I collagen (RTCol, Merck, Hertfordshire, UK; [82]), bovine collagen Type II (BCol, Sigma Aldrich, Dorset, UK; [83]) and bovine gelatin (BGel, Sigma Aldrich) which is thermally denatured collagen that has been used to form scaffolds for biomaterial research [84].

### 4.2. Collagen Scaffold Fabrication

*R. pulmo* collagen solution (4 mg/mL in 0.1 M acetic acid) was used for scaffold preparation. Each collagen solution (jellyfish/rat-tail/bovine and bovine gelatin) was frozen at −20 °C in a 96 well plate and then freeze-dried. The constructs were then chemically crosslinked with 1-ethyl-(3-3-dimethylaminopropyl) carbodiimide hydrochloride (EDC; 1% *w*/*v* in 80% ethanol) for 90 min, rinsed with deionsed water and incubated with 1% glycine for 16 h. The constructs were then rinsed again in deionised water before being freeze-dried again.

### 4.3. Scanning Electron Microscopy Scaffold Characterisation

Scaffold samples were rinsed (×3) in 50 mM sodium cacodylate-HCL buffer solution (pH 7.2–7.4) at 10 to 20 min intervals, before being fixed overnight in 2% glutaraldehyde and dehydrated with a graded ethanol series (30–100%). Dehydrated samples were then rinsed in 50% hexamethyldiasilazane (HMDS)/ethanol solution for 10 min, before further rinsing in 100% HDMS (×3) and left overnight to dry. The samples were then coated in chromium (15 nm) using sputter coating and were imaged using a S4800 scanning electron microscopy (SEM, 10 kV, Hitachi, Oxon, UK). Average scaffold pore size was calculated by measuring the widest point of pores within the scaffold structures using proprietary Hitatchi SEM software. A total of 30 pores were analysed from each separate fabrication run (n = 3).

### 4.4. Differential Scanning Calorimetry Characterisation

Scaffolds were placed in platinum crucibles and heated in a STA 449F1 Thermal Analyser (Netzsch, Wolverhampton, UK). Heating and cooling were performing in flowing argon gas with a temperature ramp of 10 °C min^−1^ from room temperature to 100 °C and an empty platinum crucible was used for reference.

### 4.5. Collagenase Digest Assay

Scaffolds were placed into tubes containing 3 mL Dulbeccos Modified Eagle Medium (DMEM, Gibco, Thermo Fisher Scientific, Altrincham, UK) supplemented with 300 U mL^−1^ bacterial collagenase and incubated at 37 °C on a tube rotator (Miltenyi Biotec, Surrey, UK). At given time points (0–12 h) scaffolds were removed, blotted dry and weighed before being returned to collagenase solution.

### 4.6. Protein Sequencing and Bioinformatics

Specific bands of interest were excised from the gel and sent to Proteome Factory (Berlin, Germany) for peptide identification through liquid chromatography mass spectrometry. The peptide sequences were placed in basic local alignment search tool (BLAST) and Clustal Omega (a multi sequence tool) for specific human FASTA collagen sequence alignment to jellyfish peptides. Functional peptide analysis was conducted using Abdesigner where human FASTA sequence (COL1A1 and COL3A1) was input and graphical output was used to inform functionality of areas with high sequence similarity to jellyfish (*R. pulmo*) peptides from multi sequence alignment.

### 4.7. miRNA Detection in Collagen

Detection of miRNA was performed by Sistemic Biomedical Company (Glasgow, UK). Four samples of Jellagen-purified *R. pulmo* jellyfish collagen and four samples of bovine collagen (Sigma Aldrich) were tested. Total RNA was isolated from each sample and assessed for concentration and purity at absorbance ratios at 260/280 nm and 260/230 nm. Samples were analysed on the Agilent miRNA platform (SurePrint G3 Human v21 microRNA 8 × 60 K microarray slides, Agilent, Cheshire, UK). An RNA input amount of 100 ng, from a working solution at 50 ng/µL in nucleasefree water, was used for each microarray experiment. Each slide contains 8 individual arrays, with each array representing 2549 human miRNAs, where RNA labelling was performed with single-colour, Cy3-based reagent.

### 4.8. Whole Blood Collection

Peripheral blood was collected from healthy adult volunteers (n = 3) into lithium heparin Vacutainer (Vacuette^®^) tubes. All blood samples were collected with informed written consent and study approved by Wales Research Ethics Committee 6 (13/WA/0190).

### 4.9. Platelet Activation Assay

Whole blood was stained with 200 ng/μL CD42b-FITC (clone HIPO, IgG1, eBioscience, Thermo Fisher Scientific, Altrincham, UK), before the blood was vortexed, incubated and lysed using BD FACS lysing solution. Whole blood was either unstimulated or stimulated with 4 mM phorbol 12-myristate 13-acetate (PMA) for 20 min as a positive control. AbC beads (Life Technologies, Thermo Fisher Scientific, Altrincham, UK) were stained with the antibody and used for compensation. Within 2 h of staining, samples were acquired on a flow cytometer (violet: 405 nm, blue: 488 nm, red: 638 nm lasers, Navios, Beckman Coulter, High Wycombe, UK) using linear forward scatter (FSC) versus side scatter (SSC) scale, with flow rate set to high. Samples were acquired on a logarithmic scale with 10,000 events as the stopping gate. Compensation and data analysis were performed using Kaluza 1.3 (Beckman Coulter, Beckman Coulter, High Wycombe, UK).

### 4.10. Leukocyte Activation and Death

Whole blood was stained with the following: CD15 and CD14; 100 ng/µL CD3-APC-AF750 (clone UCHTI, IgG1, Backman Coulter); 25 ng/µL CD62L-PE (clone DREG-56, IgG, eBioscience, Thermo Fisher Scientific, Altrincham, UK); 100 ng/μL CD11b-APC (clone CBRMI/5, IgG, eBioscience); 220 ng/μL fMLP receptor-FITC (clone REA169, IgG, Miltenyi Biotec). The blood was then vortexed, incubated and lysed using EasyLyse (Epicentre^®^ Biotechnologies, Cambridge, UK), before 1 μL DRAQ7, final concentration 20 µM (BioStatus, Leicestershire, UK), was added. Whole blood was left unstimulated or stimulated with 10 ng/mL LPS for 4 h (37 °C, 5% CO_2_) as a positive control (data not shown) and unstained samples were used for gating purposes. Blood exposed to 1% Triton-X 100 was used to determine DRAQ7 positive cell gates. Flow cytometry was performed as described in Section 4.9, with stop gate on 10,000 CD15^+^ events which would allow for the acquisition of approximately 10,000 total lymphocytes and 1000 monocytes.

### 4.11. Cytokine Release Profile Assay

Levels of IL-6 and IL-10 in cell culture supernatants from whole blood cultures were measured using specific enzyme-linked immunosorbent assays (ELISAs) in accordance with manufacturer’s instructions (Duo-Set, R & D Systems, Abingdon, UK), using capture antibodies and biotinylated secondary detection antibodies. The optical density of each well was determined using the FLUOstar Omega plate reader (BMG Labtech, Bucks, UK) at an absorbance of 450 nm.

### 4.12. Bovine Articular Cartilage Tissue Isolation

Full depth immature (7–14 days) bovine articular cartilage samples were obtained from metacarpophalangeal joints (n = 3). Full thickness cartilage tissue was harvested from the femoral and lateral condyles using a 6 mm biopsy punch.

### 4.13. Bovine Chrondroprogenitor Cell Isolation

Harvested cartilage was placed in low glucose DMEM (ThermoFisher Scientific, Thermo Fisher Scientific, Altrincham, UK) before the cells were isolated from the whole tissue sample by sequential digestion in pronase in DMEM solution (70 U mL^−1^, 2 h, 37 °C) on a tube rotator before the tissue was resuspended in collagenase (300 U mL^−1^, 16 h, 37 °C). Cells were then collected after passing the digest through a 40 µm cell strainer before cell counting on an automated cell counter (Bio-Rad, Hertfordshire, UK).

Chondroprogenitor cells have been shown to be selected through the differential fibronectin assay [48]. Six-well plates were coated with 10 µg mL^−1^ fibronectin in 0.1 M phosphate buffered saline (PBS, pH 7.4) containing 1 mM MgCl_2_ and 1 mM CaCl_2_ at 4 °C for 24 h. Isolated full depth cells (1000 cell mL^−1^) were seeded onto coated plates for 30 min at 37 °C in DMEM. After 20 min, media and non-adherent cells were removed. Fresh culture media consisting of DMEM (Gibco, Thermo Fisher Scientific, Altrincham, UK), 1% penicillin and streptomycin, 4.5 g mL^−1^ L-glucose, 10 mM HEPES, 1 mM sodium pyruvate, 2 mM L-glutamine and 10% foetal bovine serum (FBS) was added to the adherent cells before further incubation for 6 days prior to colony isolation and monolayer expansion

### 4.14. Scaffold Cell Seeding

Scaffolds were placed in 24-well plates coated in 2% agarose to prevent cell attachment and equilibrated in culture media for 10 min. Bovine chondroprogenitor cells (5 × 10^5^) were resuspended in 80 µL of media before seeding onto the surface of the scaffold structure and incubating for 20 min (37 °C and 5% CO_2_) to allow cell attachment before filling each well with a further 200 µL of media. After 24 h, culture media was changed to chrondrogenic media and refreshed every 2 days for 21 days. Chrondrogenic media consisted of high glucose DMEM (Gibco), supplemented with 10% heat inactivated FBE, 1% insulin transferring selenium (ITS) and 1% penicillin and streptomycin, with/without 10 ng/mL TGFβ1.

### 4.15. Histology of Scaffolds and Native Bovine Tissue Seeded with Chrondroprogenitor Cells

Tissue and scaffolds samples seeded with bovine chondroprogenitor cells were washed in PBS before being fixed in 4% neutral buffered formalin with saline (NBFS) and PBS washed again. Samples were dehydrated through a graded ethanol series (70%, 95% and 100% × 2) before being cleared in xylene for 20 min and infiltrated with paraffin wax for 1 h at 56 °C. The wax embedded samples were sectioned into 7 μm thick slices using a microtome and transferred onto a poly-l-lysine histology slide. Prior to staining, the samples were deparaffinised by washing (×2) in xylene for 2 min and rehydrating in a series of graded ethanol (100% × 2, 95% and 75%). The samples were then stained to identify cellular infiltration (haemotoxylin 33% and eosin 1%; 1–2 min incubation), proteoglycan content (toluidine blue 0.04%, 15 min incubation) and collagen organisation (picrosirius red 0.1%, 1 h incubation). For haemotoxylin and eosin and picrosirius red staining, the samples were washed in water and dehydrated in ethanol before being mounted with DPX mounting media, while toluidine blue samples were washed with water and dried overnight at 45 °C before being mounted with DPX and before viewing under a light microscopy.

### 4.16. Biochemical Analysis of Chondrogenesis

Bovine chondroprogenitor cells were seeded in collagen scaffolds and cultured in chondrogenic media for 21 days before the scaffolds were digested in papain digestion buffer (20 mM NaAc, 1 mM EDTA, 2 mM DTT, 300 μg/mL papin) at 60 °C until no visible tissue remained. Total DNA content was quantified using Quant-iT PicoGreen dsDNA kit assay (Life Technologies, Thermo Fisher Scientific, Altrincham, UK) and compared to a standard curve composed of known concentrations of lambda DNA diluted in 1× TE buffer (0–10 μg/mL). Fluorescence was measured (485 nm excitation; 520 nm emission) by a spectrofluorometer (FLUOstar Omega, BMG Labtech, Bucks, UK).

Total glycosaminoglycan (GAG) content was quantified using dimethylmethylene blue (DMMB) assay. 20 µL of papain-digested sample was added to 200 µL of DMMB reagent (16 mg L^−1^ dimethlmethylene blue, 3 g poly vinyl alcohol, 3.04 g glycine, 2.37 g NaCl, 95 mL 0.1 M HCl) in a 96-well plate and shaken for 5 min. Absorbance was measured at 525 nm using a plate reader (FLUOstar Omega, BMG) against a standard curve with known concentration of chondroitin sulphate (0–10 μg/mL) and normalised through division by the corresponding DNA content.

Hydroxyproline assays were run to calculate collagen content. Papain digested samples were hydrolysed in 6 M HCl for 24 h at 110 °C and vacuumed dried overnight before reconstituting in deionised water. Following centrifugation to remove impurities, 30 µL of each sample were added to each 96-well plate and 70 µL of diluent solution (100 mL propan-2-ol, 50 mL H_2_O) and 50 µL oxidant (0.7 g chloramine T, 10 mL H_2_O and 50 mL of stock buffer consisting of 28 g sodium acetate trihydrate, 18.75 g tri sodium citrate dihydrate, 2.75 g citric acid, 200 mL propan-2-ol) were added to each sample before shaking for 5 min. 125 µL of colour reagents (7.5 g dimethylaminobenzaldehyde, 11.25 mL perchloric acid (60%), 62.5 mL propan-2-ol) were added and samples shaken prior to 15 min incubation at 70 °C. Hydroxyproline content was measured by absorbance at 540 nm using a plate reader (FLUOstar Omega, BMG) and quantification determined against the standard curve of trans-4-hydroxy-l-proline (0–100 µg/mL). hydroxyproline content were normalised against DNA content.

## Data Availability

Data generated and analysed during this study are included in this published article and its Supplemental information file. Additional details are available upon reasonable request.

## Supplementary Materials

The following are available online at https://www.mdpi.com/article/10.3390/md19080405/s1, Figure S1: AbDesigner output for human collagen genes. Figure S2: Stability of RTCol and BGel scaffolds.
