# Supplementary material for: Jellyfish Collagen: A Biocompatible Collagen Source for 3D Scaffold Fabrication and Enhanced Chondrogenicity
Source: Mar Drugs. 2021 Jul 22;19(8):405. doi: 10.3390/md19080405 (PMC8400217; doi:10.3390/md19080405)
Supplement: Supplementary file 1 [file marinedrugs-19-00405-s001.zip › marinedrugs-1272971-supplementary.pdf]

marine drugs

MDPI

Article

Jellyfish Collagen: A Biocompatible Collagen Source for 3D Scaffold Fabrication and Enhanced Chondrogenicity

Zara Ahmed[1]†, Lydia C. Powell[1]†, Navid Matin[1], Andrew Mearns-Spragg[2], Catherine A Thornton[1], Ilyas M. Khan[1] and Lewis W. Francis[1,*]

COL1A1

(a)

(b)

COL3A1

(c)

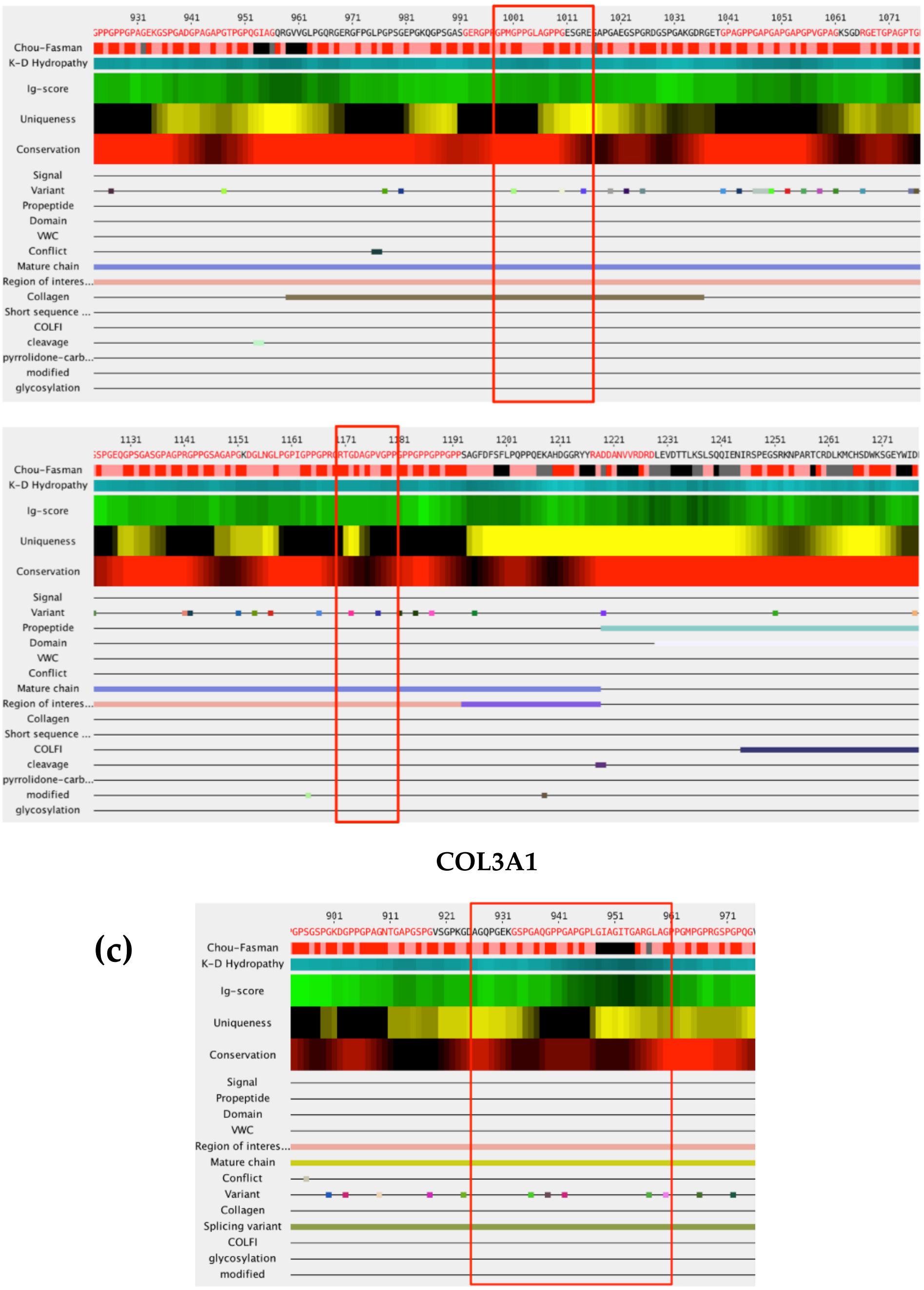

Figure S1: AbDesigner output for human collagen genes. Human COL1A1 positions (a) 998 to 1115 and (b) 1170 to 1180 and human COL3A1 positions (c) 926 to 960 demonstrate the functional relevance of regions of high similarity with jellyfish collagen peptides.

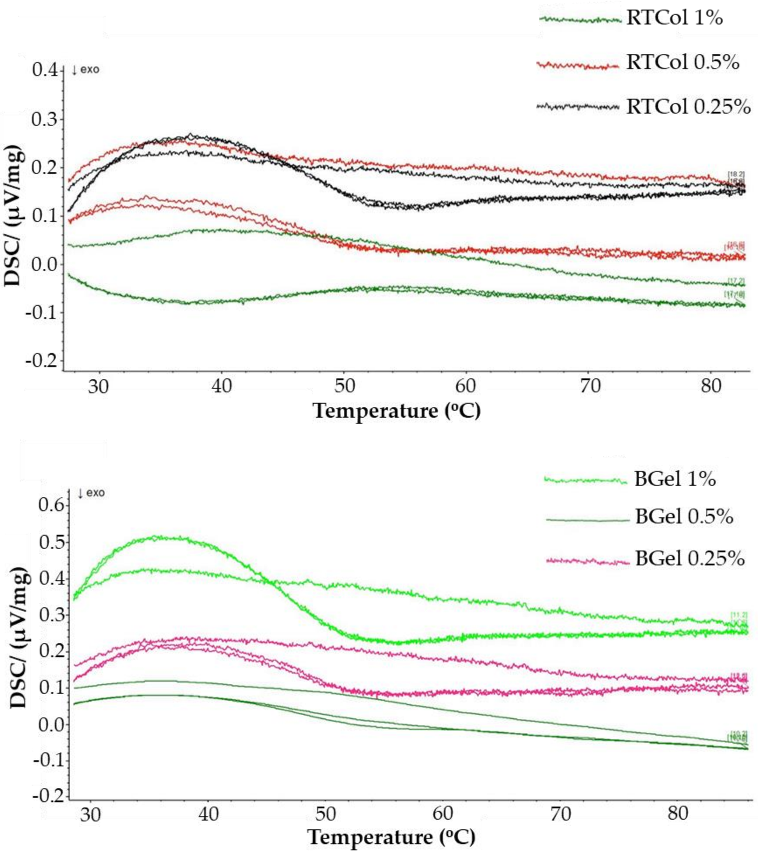

Figure S2. Stability of RTCol and BGel scaffolds. Differential scanning calorimetry (DSC) of RTCol and BGel scaffolds with 0.25%, 0.5% and 1% EDC crosslinking treatment.
